# Meta-Analysis of the Related Nutritional Supplements Dimethyl Sulfoxide and Methylsulfonylmethane in the Treatment of Osteoarthritis of the Knee

**DOI:** 10.1093/ecam/nep045

**Published:** 2011-02-17

**Authors:** Sarah Brien, Phil Prescott, George Lewith

**Affiliations:** ^1^Department of Primary Medical Care, University of Southampton, Aldermoor Health Centre, Aldermoor Close, Southampton, Hampshire, SO16 5ST, UK; ^2^School of Mathematics, University of Southampton, Southampton, Hampshire, SO16 5ST, UK

## Abstract

Dimethyl sulphoxide and methylsulfonylmethane are two related nutritional supplements used for symptomatic relief of osteoarthritis (OA). We conducted a meta-analysis to evaluate their efficacy in reducing pain associated with OA. Randomized or quasi-randomized controlled trials (RCTs), identified by systematic electronic searches, citation tracking and searches of clinical trial registries, assessing these supplements in osteoarthritis of any joint were considered for inclusion. Meta-analysis, based on difference in mean pain related outcomes between treatment and comparator groups, was carried out based on a random effect model. Seven potential trials were identified of which three RCTs, two DMSO and one MSM (total N = 326 patients) were eligible for inclusion. All three trials were considered high methodological quality. A significant degree of heterogeneity (*χ* ^2^ = 6.28, *P* = .043) was revealed. Two studies demonstrated statistically significant (but not clinically relevant) reduction in pain compared with controls; with one showing no group difference. The meta-analysis confirmed a non significant reduction of pain on visual analogue scale of 6.34 mm (SE = 3.49, 95% CI, −0.49, 13.17). The overall effect size of 1.82 was neither statistically nor clinically significant. Current evidence suggests DMSO and MSM are not clinically effective in the reduction of pain in the treatment of OA. No definitive conclusions can currently be drawn from the data due to the mixed findings and the use of inadequate dosing periods.

## 1. Introduction

Osteoarthritis (OA) is the most common of all joint disorders and affects over 30 million people in the US and 1 in 10 people aged 35–75 in the UK [1] and is associated with pain and functional disability, which in turn leads to reduced quality of life and increased risk of further morbidity and mortality [2]. The treatment of OA is largely symptomatic and includes analgesics, NSAIDs as well as exercise and surgical intervention [3]. The long-term use of NSAIDs is associated with serious gastrointestinal side effects [4, 5] including 12 000 hospital admissions and ~2000 deaths in the UK every year [6]. Patients with OA turn to complementary and alternative medicine (CAM) to gain symptomatic relief and avoid iatrogenic illness with OA being the sixth most common condition treated with CAM [7]; OA patients use of CAM is substantially greater that in the general population with a reported prevalence of up to 90% [8, 9].

Two nutritional supplements, dimethyl sulfoxide (DMSO, an organic form of sulfur commercially prepared from lignin) and its oxidized form, methylsulfonylmethane (MSM, occurring in green plants fruits and vegetables) have been used to treat arthritic conditions [10]. DMSO is converted in the body to MSM and as MSM remains in the body for longer than DMSO [11], it is suggested that many of the beneficial effects of DMSO are due to the long lasting fraction of DMSO which is converted to MSM [12]. Both have similar pharmacological properties and their putative effects and mechanisms have been reviewed previously (MSM [13–15]; DMSO [16–19]; both [20, 21]). MSM and DMSO have been reported to reduce peripheral pain [22–24], inflammation [25] and arthritis [26], and might inhibit the degenerative changes occurring in OA [27]. These compounds may act through their ability to stabilize cell membranes, slow or stop leakage from injured cells and scavenge hydroxyl free radicals which trigger inflammation [23, 25, 28–33]. Their sulfur content may also rectify dietary deficiencies of sulfur improving cartilage formation [34, 35].

DMSO is a topical agent, diluted for therapeutic use (concentrations are expressed %(v/v)). It penetrates the skin and it is also used as a carrier to aid penetration of other medications [24, 28, 36]. Clinicians are advised to prescribe DMSO for OA for at least three months to ensure a clinical effect. However the optimum dosage for this supplement in OA has not been clearly evaluated as no dose ranging studies have been conducted. Previous empirical reports suggest that the therapeutic concentrations of DMSO are 60%–90% [18, 37] and that doses under 10% are probably clinically inactive [37–39]. There is limited formal safety data and no long-term assessment of DMSO although the toxicity of oral DMSO appears very low (LD50 = 14.5 g/kg body weight). Adverse effects associated with topical DMSO administration have been reported (GI upset, skin irritation, and garlic like taste, breath and body odour) [40, 41]. Its garlic odour can compromise blinding in double blinded trials.

MSM is used orally and topically. Like DMSO, the treatment duration for OA is at least three months. The optimum dosage has not been clearly defined as no dose ranging studies have been carried out. The suggested oral therapeutic doses is 4–6 g per day [42, 43], although doses of up to 20 g/day have also been used [44]; over the counter preparations are typically 1–5 g daily [45]. There is limited formal safety data and no long term assessment. However, MSM is rapidly excreted from the body [46, 47] and animal toxicity studies of MSM showed only minor adverse events using doses of 1.5 g/kg and 2.0 g/kg of MSM for 90 days. This dose represents a human dose of 105–140 g/day, which is equivalent to 17–23 times the proposed maximum recommended human dose of 6 g/day [48]. A further study confirmed MSM had no toxic effects on either pregnant rats or their foetus [49]. Only minor adverse effects are associated with MSM administration in humans and include allergy, GI upsets and skin rashes [50].

Two systematic reviews have also been conducted on these supplements. Ameye and Chee [13] conducted a systematic review of a range neutriceuticals in OA which included MSM and concluded that MSM showed “moderate” evidence of efficacy. Brien et al. [21] reported that current evidence precludes definitive conclusions about the efficacy of either supplement but recommended further investigation addressing methodological concerns including optimal dosage and treatment duration. A meta-analysis to assess the most precise estimate of the efficacy of these supplements is therefore timely given their frequent use [8, 9] and pertinent because of the withdrawal of some COX-2 inhibitors [5].

## 2. Methods

### 2.1. Literature Search Strategy

Searches were performed using the following electronic databases to identify relevant studies available for inclusion in the meta-analysis; Cochrane Library (1970–2008), MEDLINE (1950–2008), EMBASE (1980–2008), AMED (1985–2008), CINAHL (1982–2008), SCOPUS (1996–2008) and the National Library for Health (Complementary and Alternative Medicine Specialist Library). Free text searches were performed on each database with the following keywords: osteoarthritis, degenerative joint disorder, dimethyl sulfoxide, DMSO, methylsulfonylmethane, MSM, clinical trial: double blind; single blind; RCT; placebo; randomized; comparative study; evaluation study; control. The search strategies for dimethyl sulfoxide and methylsulfonylmethane are shown in Tables 1 and 2, respectively.

Citation tracking was undertaken to identify unpublished trials. As numerous pharmaceutical companies market DMSO, it was impractical to contact each of them for unpublished data. Finally we additionally searched four clinical trials registries (http://www.clinicaltrials.gov/; http://www.controlled-trials.com/; http://www.actr.org.au/; and http://www.umin.ac.jp/ctr/), to identify ongoing trials. The last update of searches was performed in June 2008.

### 2.2. Trial Selection

All articles that reported randomized or quasi-randomized controlled trials (RCTs) comparing oral or topical formulations of DMSO or MSM in the treatment of OA were identified. As current data shows that using greater than 10% DMSO [35–37] and over 1 g/day MSM [45] are needed for therapeutic effects only RCTs assessing at least these minimal levels were included. RCTs were included if they were; in humans; reported comparison of DMSO or MSM to placebo; used validated outcome measures for OA; and did not include patients with other joint pathology.

### 2.3. Data Extraction

Data was extracted independently by two of the authors (S.B. and P.P.). Data regarding publication status, trial design, patient characteristics, treatment regimens, outcome measures, results and findings were extracted. Effect sizes were calculated where appropriate. Where information was insufficient the authors were contacted to request missing information. Disagreements were resolved by discussion by all three reviewers and subsequent consensus.

### 2.4. Quality Assessment

Two reviewers (S.B. and P.P.) independently assessed the trial quality using the JADAD [51] scale to assess randomization, blinding and withdrawal. The total JADAD score is 5 and studies were considered low quality if they reported a score less than 3, or high quality for scores 3–5. Additional quality assessment was also conducted including assessment of concealment of treatment allocation. Assessment of internal and external validity for these papers have also been reported elsewhere [21]. Disagreements were resolved by discussion with the third author.

### 2.5. Data Synthesis

The selected outcome measure for analysis was the measurement of pain, as this is the most patient relevant outcome in this condition. The primary outcome measure was mean change in pain from baseline. Pain levels were assessed using either a 100 mm visual analogues scales (VAS) and on the Western Ontario MacMaster Osteoarthritis Index (WOMAC) pain subscale [52]. As the WOMAC subscales have a 0–20 score, these are adjusted up to a 0–100 range for comparability. These were used to assess the differences between the intervention groups (MSM or DMSO) and the control groups (placebo or standard conventional treatment, diclofenac). Data points were chosen at the end of the treatment period.

### 2.6. Statistical Analysis

Summary estimates of the treatment effect were calculated using the weighted means of the within study treatment effects [53]. Weighted mean differences and 95% confidence intervals (CI) were calculated using both a fixed and random effects model. Where data were insufficient the original authors were contacted to request the missing information. Sensitivity analyses were conducted where appropriate. Effect sizes were calculated by dividing the difference in the changes, from baseline to the end of the trial period, between treatment groups by the estimated standard error. An effect size of about 0.8 is considered minimally clinically relevant [54]. Chi square test was used to investigate the heterogeneity in the treatment difference parameter across the studies. Publication bias was explored by using a funnel plot, whereby effect estimates of the pain were plotted against trial sample size. The funnel plot was examined visually but due to the small number of studies it was not feasible to statistically test for symmetry.

## 3. Results

### 3.1. Three RCTs Met Inclusion Criteria

A total of seven citations were retrieved from the databases that met the inclusion criteria [36, 42, 43, 55–58]. Two further clinical trials were identified from citation tracking [39, 59]. No further studies were identified from searching clinical trials registries. Citations were excluded if: they were not randomized placebo controlled trials; they reported an intervention other than DMSO/MSM; assessed another treatment condition; or they did not assess either DMSO or MSM.

Seven double blind, placebo controlled, randomized trials, assessing either DMSO or MSM in the treatment of OA in our literature search which met the inclusion criteria were identified for possible inclusion [36, 39, 42, 43, 56, 57, 59]. Of these, three were eligible for inclusion in the meta-analysis. Four trials were excluded because either (i) DMSO was not specifically assessed as a therapeutic agent [57, 59]; (ii) there was incomplete data [42] (mean and SD for placebo group were not reported in the text and attempts to contact the authors were unsuccessful); or (iii) the pain scale was assessed using a Likert scale rather than VAS [39] and hence continuous data could not be extracted for the analysis. Only selected data from one study [36] were included; this study had three intervention arms (diclofenac versus DMSO versus placebo) so only the arms relating to the assessment of DMSO versus placebo were included. A search of trial registries yielded no ongoing trials. Therefore the final analysis included three studies, one of which assessed MSM [43] and two DMSO [36, 56].

### 3.2. Description of Studies

Table 3 describes the main study characteristics of the studies included in the meta-analysis. Further study details relating to the studies included in this meta-analysis can be obtained from our recent review of all clinical trials assessing DMSO/MSM in OA [21]. Overall, the trials allocated 326 patients of which 161 received active treatment (DMSO, *N* = 136 or MSM, *N* = 25). All three trials assessed patients with OA of the knee joint. Two trials assessed the supplement DMSO topically administered [36, 56] and one MSM, oral administration [43]. All trials assessed these supplements as an alternative rather than adjunctive treatment for OA of the knee, and rescue medication was allowed. The average mean age of the patients included in these trials ranged from 56 [43] to 62 years [36, 56]. 

#### 3.2.1. Baseline Characteristics

Baseline demographic characteristics were reported in all studies and all showed no significant differences between groups. However, the data describing the characteristics of the patients entered into these three trials was not fully reported by the trials. The average duration of OA was reported in only the Kim study (mean duration of arthritis was 6 years); although patients were included in the Bookman and Eberhardt studies only if they had radiological diagnosed OA for a minimum of 6 months or 5 years, respectively. The severity of OA of included patients was reported in only one trial [43] which stated 95% of patients entered had low to moderate OA (i.e., Kellegren-Lawrence grades between 0 and 2).

#### 3.2.2. Study Medication

All studies assessed the supplements as alternative treatments with all concurrent systemic and topical medications stopped prior to commencement of the study. The use of rescue medication was reported in two of the studies [36, 43]. The dosage and treatment duration of the supplements in these trials has been previously reviewed and highlighted concerns about inadequate treatment duration and dosage of these supplements assessed in clinical trials [21]. The doses employed by all three trials assessed in this meta-analysis were all below current recommendations. Duration of treatment was also inadequate in two (the DMSO trials [36, 56]) of the three trials; the Kim trial assessed MSM for an adequate duration. Compliance assessment was reported in two of the studies ([43] pill count; [36] weighing medication bottles). No follow up assessment was performed in any of the three trials.

#### 3.2.3. Pain as the Primary Outcome

Pain was assessed as the primary efficacy outcome in all these trials. Pain was assessed either by 0–100 mm VAS [56] or the WOMAC pain subscale [36, 43]. Those studies reporting pain via the WOMAC pain scales either assessed this using or 0–100 scale [43] or a 0–20 scales [36]; the latter results were scaled up to a 0–100 range for comparability.

The Eberhardt study [56], although it included more patients than the other studies, did not provide any *a priori* power calculations. However, the observed difference between treatment groups in reduction of pain from baseline to end of treatment of 11.7 mm was shown to be statistically significant, *P* = .0019; and our sensitivity analysis confirmed power was adequate, that is, greater than 90%. Bookman et al. [36] calculated that 50 patients per group, including allowing for dropouts, would be required for an 80% power to detect a specified difference between the two treatment arms and a previous analysis confirmed power was adequate [21]. Kim et al. [43] included a power calculation for the improvement from baseline to 12 weeks, claiming that 25 patients per group would be sufficient to detect with 80% power an improvement in pain score of 25%; however this was not based on the difference between treatment groups. Our subsequent sensitivity analysis confirmed this study had a power of 55% to detect a difference between treatment groups at 5% level of significance.

#### 3.2.4. Trial Quality Was High

Table 4 reports the methodological quality characteristics of the included trials. All three trials were of high quality, scoring JADAD scores of either 4 [56] or 5 [36, 43]. All trials were reported as being randomized. The allocation sequence was reported as being adequately generated in two of the trials [36, 43] but no details were provided in the Eberhardt study regarding the randomization process [56]. All the trials were adequately double blinded with the reported use of identical dummy tablets (MSM) or dummy gel (DMSO). They all described withdrawals and exclusions and analyzed all randomly assigned patients and hence conducted an intention to treat. Two of the trials [36, 56] reported their approach for handling missing data; using last observation carried forward for imputations [60].

#### 3.2.5. Meta-Analysis—Effect on Joint Pain

**Figure 1:**
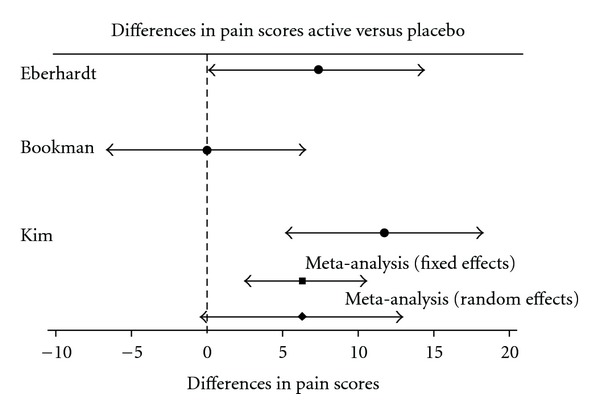
Meta-analyses of randomized controlled trials.

Key data from the included trials is presented in Table 5. Two of the trials [43, 56] reported mean differences that favour DMSO/MSM over placebo with effect sizes of 3.53 and 2.06, respectively; and both trials show 95% CI that do not overlap zero indicating significant differences. Bookman et al. [36] identified no group differences in pain reduction (effect size = 0.0). The initial fixed effects meta-analysis of all the trials indicates a statistically significant reduction of pain scores of 6.3 mm (CI: 2.41, 10.27) from baseline in favour of DMSO/MSM compared to placebo (Figure 1). However this change is not clinically significant; a significant improvement of 9–12 mm on WOMAC subscales in pain, is considered clinically relevant [61–63] as is a >17.5 mm change [64] in VAS pain scales.

A chi-squared test of homogeneity was just significant (*χ* ^2^ = 6.28, *P* = .043), suggesting that the effects are more variable than would be expected by chance and indicating inconsistencies in the results of the studies. A random effects meta-analysis identified a slight increase in SE of the mean difference of 6.3 mm with SE = 3.49S and 95% CI, (−0.49 to 13.17) which is not quite statistically significant. The funnel plot also confirmed asymmetry in the results, due to the Bookman et al. study [36] to the left of the common effect indicating the possibility of publication bias.

## 4. Discussion

The findings of this meta-analysis suggest that based on available evidence, DMSO/MSM leads to an overall reduction in pain VAS of 6.34 mm compared to placebo. This is not statistically significantly superior to placebo in reducing pain associated with OA of the knee. Results from the Bookman trial [36] showed no difference in effect between treatment and placebo which is in contrast to the results from the other two trials; this may be due to a larger than anticipated placebo response or may reflect possible publication bias.

Clinical significance is critical for meaningful meta-analyses [65] designed to assist clinicians in their decision making. Current guidelines suggest a minimal clinically important improvement (MCII) for the pain subscale for WOMAC in patients with knee OA requires a minimum change of 19.9 mm from baseline to end of treatment [61]; whilst the MCII for VAS of pain in this condition is a minimum of 17.5 mm [64]. It has also been observed that baseline pain levels affect patient perceived clinical significance. Recommendations have been proposed for future trials that are based on reporting MCII specific to baseline severity levels [61]. Pain score reductions from baseline to end of treatment, in those receiving active treatment with DMSO were 12.5 mm ([36]; WOMAC pain subscale) and 42.7 mm ([56]; VAS); and 14.6 mm ([43]; WOMAC pain subscale) for patients receiving MSM. Based on current guidelines, only those patients in the Eberhardt study (both active and placebo treated) reported clinically meaningful reductions in their pain and showed a reduction in pain levels comparable with standard conventional treatment with COX 2 inhibitors such as Rofecoxib (35.4 mm decrease [66]; 28.1 mm [67]). This meta-analyses confirms that based on the current limited evidence these nutritional supplements are neither statistically nor clinically more effective than placebo in reducing pain in knee OA. 

### 4.1. Strengths and Limitations

Only randomized, double blind, placebo controlled trials were included in this investigation. Our review is based on a systematic and thorough literature search. It is unlikely we missed relevant trials data but as possible data was not sought from pharmaceutical companies this cannot be ruled out. Data extraction, including quality assessment, was completed independently by two authors to minimize bias. The reporting quality of the trials was acceptable and the quality assessment tool is validated and its components are associated with bias. All three studies assessed OA of the knee hence enabling direct comparison between trials.

There are some limitations of this meta-analysis. First, only three trials were available for inclusion assessing *N* = 326 patients in total, of which *N* = 161 received active treatment. Further trials may therefore affect the future assessment of these supplements. In addition specific methodological concerns have been identified in these trials including the issue of adequate treatment period with only one study assessing the supplements for a clinically relevant time [43].

### 4.2. Implications for Research and Clinical Practice

Recent reviews ([21] DMSO and MSM; [13] MSM)) of these supplements for OA suggest further studies of these nutritional supplements may be warranted if the study design takes into account the methodological issues identified in this article. This meta-analysis does not currently provide robust evidence to support the use of DMSO or MSM for OA however the conclusions are affected by the number and quality of included trials. The three trials assessed had good methodological quality and adequate reporting yet significant methodological concerns about dosing and treatment duration temper any definitive conclusions. In addition, due to the paucity of studies of these supplements, the publication of further trials may have a major effect on these recommendations.

If we wish to further evaluate these supplements then large scale trials are needed that are adequately powered and assess patients for a clinically relevant duration using optimal dosage. Future trials should also report the minimal clinically important improvement with reference to the patient's baseline severity [61]. A search of clinical trial registries revealed no ongoing trials and it seems unlikely that suitable evidence investigating the effectiveness of these supplements will become available in the near future. The implications for current clinical practice are that these supplements are not specifically effective in decreasing the pain associated with OA knee. Given the additional lack of safety reporting data as previously described in a recent systematic review [21], it is also not possible to have clarity about their safety.

## Figures and Tables

**Table 1:** Search strategy for DMSO.

(1) Explode “osteoarthritis”/all subheadings
(2) Explode “degenerative arthritis”/all subheadings
(3) Osteoarthr$
(4) (1)–(3)
(5) Dimethyl sulfoxide
(6) DMSO
(7) (5)–(6)
(8) Clinical trial
(9) Double blind
(10) Single blind
(11) RCT
(12) Placebo
(13) Randomised
(14) Comparative study
(15) Evaluation study
(16) Control
(17) 8 OR 9 OR 10 OR 11 OR 12 OR 13 OR 14 OR 15
(18) 4 AND 7 AND 17
(19) LIMIT 18 to HUMAN

**Table 2:** Search strategy for MSM.

(1) Explode “osteoarthritis”/all subheadings
(2) Explode “degenerative arthritis”/all subheadings
(3) Osteoarthr$
(4) (1)–(3)
(5) Methylsulfonylmethane
(6) MSM
(7) (5)–(6)
(8) Clinical trial
(9) Double blind
(10) Single blind
(11) RCT
(12) Placebo
(13) Randomised
(14) Comparative study
(15) Evaluation study
(16) Control
(17) 8 OR 9 OR 10 OR 11 OR 12 OR 13 OR 14 OR 15
(18) 4 AND 7 AND 17
(19) LIMIT 18 to HUMAN

**Table 3:** Main study characteristics.

Author	Study design	Joint location	Sample size	Intervention/control	Outcome measures	Main result
Eberhard et al., 1995 [56]	Double-blind, placebo-controlled	Knee	112 (56 DMSO; 56 placebo)	25% DMSO gel 5–8 cm Placebo gel 5–8 cm Dosage: TID Treatment period: 3 weeks	Primary outcome: change in pain scores for. (i) Pain under loading VAS(ii) Pain at rest: Likert scale(iii) Pain on palpation: Likert scaleSecondary outcomes: (i) Mobility six-point likert(ii) Swelling(iii) Pt and physicians global assess of efficacy and tolerability(iv) Adverse events Evaluated baseline, 7, 14 and 21 days	All primary efficacy criteria significantly better than placebo Statistically significant and clinically relevant reduction in loading pain of mean 42.7 mm (reduction of 64.5%) in DMSO compared to a significant reduction of 30.8 mm in the placebo (reduction of 46.5%).Difference in mean reduction was 11.7 mm (CI −18.35 to −5.1). DMSO significantly better than placebo (*P* = .019)Pain at rest is significantly reduced by mean of −1.3 in DMSO compared to −0.9 in placebo, *P* = .015Pain on palpation is significantly reduced by mean of −1.5 in DMSO compared to −1.1 in placebo, *P* = .029NS group diff for Mobility and swelling Pt and physician global assessment better for DMSO than placebo No serious AE 9 AE DMSO; 12 P
Bookman et al., 2004 [36]	Double-blind, three arm comparative, multi-center and placebo-controlled trial	Knee	248 (84 DF; 80 DMSO; 84 placebo)	(1) Topical DF plus DMSO (45.5% wt/wt) 40 drops four times daily (2) Topical (45.5% wt/wt) DMSO 40 drops four times daily (3) Topical placebo-control solution (containing 4.5% wt/wt DMSO) 40 drops four times daily (4) Dosage: Four times a day Treatment period: QID 28 days	Primary outcomes: (i) WOMAC pain subscale Secondary outcomes:(i) Physical and stiffness subscales of (WOMAC) (ii) Weekly patient global assessment (PGA).(iii) Pain on walking (post hoc)(iv) Amount of rescue medication taken.	WOMAC pain scores was significant reduced in the DF group (−3.9 (95% (CI) −4.8 to −2.9)) compared to DMSO (−2.5 (CI −3.3 to −1.7)): *P* = .023 or placebo (−2.5 (CI −3.3 to −1.7)): *P* = .016. DF is significantly greater at reducing pain compared to DMSO and placebo. DMSO was not superior to placebo in pain reduction. DF was significantly better than DMSO and placebo for improving physical function, stiffness, pain on walking and PGA.NSD for adverse event reporting No therapeutic benefit on efficacy variables for DMSO as vehicle control (45.5%) versus placebo solution (4.5% DMSO)
Kim et al., 2006 [43]	Single centre RCT, double-blind, placebo-controlled	Knee	50 (MSM, 25; placebo *N* = 25)	(1) Oral MSM, 3 g (DMSO content <0.05%)^a^ (2) Placebo Dosage frequency: BID Treatment period: 12 weeks	*Primary outcomes* (at baseline, 2, 4, 8 and 12 weeks):(i) Pain, Physical function and stiffness and total scores (WOMAC)*Secondary outcomes (at baseline & 12 weeks)*(i) Patient and Physician GA (5-point Likert scale)(ii) SF-36(iii) Labs (CRP, homocysteine, ESR, and MDA) (iv) Use of rescue analgesia(v) weekly by telephone—compliance and AE	*Primary:* MSM significantly improved WOMAC pain (−14.6 versus −7.3, *P* = .041) & physical function (−15.7 versus −8.8, *P* = .045) compared to placebo. No significant difference noted for stiffness (−10.1 versus −6.5, *P* = .32) nor total scores (−13.4 versus −7.5, *P* = .054) compared to placebo. *Secondary:* No significant difference in decrease in either patient (*P* = .549) or physician (0.447) GA.Significant decreases in MDA (*P* = .01) and homocysteine (*P* = .004) observed in MSM compared to placebo. AE were minor (notably GI, fatigue, insomnia) and no group diff in levels reported (MSM, *N* = 21; placebo, *N* = 19). Authors reported that significant differences found in MSM were not necessarily clinically relevant when compared to NSAID treatment changes on these measures.

^*a*^One-week step up dose from 2 g per day until 6 g per day reached.

**Table 4:** Quality Assessment of RCT of MSM and DMSO randomized clinical trials.

Study, year (reference)	Baseline characteristics presented?	Concealement of allocation	Reported to be double blind	Adequate blinding of patients	Adequate blinding of researchers	Consort Diagram reported	Withdrawal rate in the intervention group	Withdrawal rate in the control group	Power Calculation/ Statistical analysis	Intention to Treat analysis performed	Method to handle missing data	JADAD Score
Bookman et al., 2004 [36]	YesNo significant group difference for demographics, baseline knee pain, radiographic status or compliance	Yes	No, but clear from methodology this was a double blind study	Yes	Yes	Yes	18% (*N* = 14/80)	18% (*N* = 15/84)	*N* = 40 per group plus 10 for drop outs, to detect a difference of 3, 4, or 5 units in WOMAC pain 80% power and *α* = 0.05 ITT analysis. Primary outcomes: analysis of covariance	Yes	LOCF Except for missing baseline data which were replaced with day 1 scores	Score = 5 *Blinding* = 2 *Randomization* = 2 *Withdrawal* = 1
Eberhardt et al., 1995 [56]	Yes No significant group differences at baseline for symptom duration, age, height, weight, length of activation of current symptoms	No details reported	Yes	Yes	No details reported	No	5% (*N* = 3/56)	2% (*N* = 1/56)	No ITT Primary outcome: ANOVA, other outcomes: contingency board method and *t*-test	Yes	LOCF	Score = 4 *Blinding* = 2 *Randomization* = 1 *Withdrawal* = 1
Kim et al., 2006 [43]	Yes No significant group differences at baseline for sex, age, height, wgt, and symptom duration, NSAID use, MSM or DMSO use, ACR classification, baseline VAS scores, PGA or PhGA scores, and radiological stage	Yes	Yes	Yes	Yes	Yes	16% *(N* = 4/25)	24% * (N* = 6/25)	Yes ITT analysis Group differences in baseline to week 12 by *T* test	Yes	Not reported	Score = 5 *Blinding* = *2 Randomization* = *2 Withdrawal* = *1 *

LOCF, last observation carried forward.

**Table 5:** Meta-analysis results.

Study	Year	Design	Analyzed patients (intervention/ placebo)	Intervention	Daily Dose	Baseline pain score (mm)	End of Rx pain score (mm)	Pain outcome measure	Mean ± SE reduction from baseline with active treatment (mm) (95% CI)	Mean ± SE reduction from baseline with placebo (mm) (95% CI)	Mean Difference ± SE in pain scores between groups (mm) (95% CI) Effect Size
Eberhardt et al. [56]	1995	Single centre Double blind placebo controlled parallel	DMSO: 56 Placebo: 56	DMSO	25% DMSO gel/day 5–8 cm	DMSO = 64.3 Placebo = 64.1	DMSO = 21.6 Placebo = 33.3	Pain VAS (0–100 mm)	42.7 ± 2.34 [38.0, 47.4]	30.8 ± 2.38 [26.1, 35.5]	11.7 ± 3.31 [5.08, 18.32] Effect = 3.53
Bookman et al. [36]	2004	Multi centre, Double blind, three arm^a^ comparative parallel arm	DMSO: 79 Placebo: 84	DMSO	45.5% wt/wt	DMSO = 46.5 Placebo = 47.0	DMSO = 34.0 Placebo = 34.5	WOMAC pain subscale (0–20 mm^b^) (scaled up to 100 mm for comparison)	12.5 ± 2.7 [7.1, 17.9]	12.5 ± 2.0 [8.5, 16.5]	0.0 ± 3.35 [−6.6, 6.7] Effect = 0.00
Kim et al. [43]	2006	Single centre, double blind placebo controlled parallel arm	MSM: 21 Placebo: 19	MSM	3 g/day oral	MSM = 58.0 Placebo = 55.1	MSM = 43.4 Placebo = 47.9	WOMAC pain subscale (0–100 mm)	14.6 ± 1.3 [12.0, 17.2]	7.3 ± 3.3 [0.7, 13.9]	7.3 ± 3.55 [0.2, 14.4] Effect = 2.06
											6.34 ± 1.96 [2.41, 10.27]
	Meta-analyses (fixed effects)										6.34 ± 3.49 [−0.49,13.17]
	Meta-analysis (random effects)										

^a^This three-armed study assessed Diclofenac, DMSO and placebo; only data for DMSO and placebo is entered. ^b^See our review paper.
